# Dr. C. Henri Leonard’s Books

**Published:** 1878-11

**Authors:** 


Dr. C. Henri Leonard's Physician's Ledger, Day-Book, and Reference and Dose-Book is received. The dose-book contains the medium and maximum doses of all officinal and many non-officinal remedies. Pocket day-book accommodates for forty families weekly, and is conveniently arranged for the purpose, while the reference and dose-book would seem to us convenient to the young physician often requiring such guide.

Physicians are invited to call and examine these books at Theodore H. Butler's book-store, 307 Main street, Buffalo.

